# Investigation of Gamma-aminobutyric acid (GABA) A receptors genes and migraine susceptibility

**DOI:** 10.1186/1471-2350-9-109

**Published:** 2008-12-16

**Authors:** Francesca Fernandez, Teresa Esposito, Rod A Lea, Natalie J Colson, Alfredo Ciccodicola, Fernando Gianfrancesco, Lyn R Griffiths

**Affiliations:** 1 Genomics Research Centre, Griffith Institute for Health and Medical Research, Griffith University, Gold Coast, Queensland, Australia; 2 Institute of Genetics and Biophysics, Adriano Buzzati-Traverso, CNR, Naples, Italy; 3 Institute of Environmental Science and Research, Wellington, New Zealand

## Abstract

**Background:**

Migraine is a neurological disorder characterized by recurrent attacks of severe headache, affecting around 12% of Caucasian populations. It is well known that migraine has a strong genetic component, although the number and type of genes involved is still unclear. Prior linkage studies have reported mapping of a migraine gene to chromosome Xq 24–28, a region containing a cluster of genes for GABA A receptors (GABRE, GABRA3, GABRQ), which are potential candidate genes for migraine. The GABA neurotransmitter has been implicated in migraine pathophysiology previously; however its exact role has not yet been established, although GABA receptors agonists have been the target of therapeutic developments. The aim of the present research is to investigate the role of the potential candidate genes reported on chromosome Xq 24–28 region in migraine susceptibility. In this study, we have focused on the subunit GABA A receptors type ε (GABRE) and type θ (GABRQ) genes and their involvement in migraine.

**Methods:**

We have performed an association analysis in a large population of case-controls (275 unrelated Caucasian migraineurs versus 275 controls) examining a set of 3 single nucleotide polymorphisms (SNPs) in the coding region (exons 3, 5 and 9) of the GABRE gene and also the I478F coding variant of the GABRQ gene.

**Results:**

Our study did not show any association between the examined SNPs in our test population (P > 0.05).

**Conclusion:**

Although these particular GABA receptor genes did not show positive association, further studies are necessary to consider the role of other GABA receptor genes in migraine susceptibility.

## Background

Migraine is a common neurological disorder with variable expression, affecting more than 12% of the general population [1]. The exact cause is unknown and there are no recognizable diagnostic pathological changes. Migraine is a neurological disorder, characterised by recurrent headache that is associated with nausea and/or vomiting, photophobia and phonophobia. The International Headache Society (IHS) has formally classified migraine into two main subtypes: migraine *with aura *(MA) and migraine *without aura *(MO) [2]. These two subtypes have substantial symptomatic overlap, but MA sufferers experience distinguishing neurological disturbances (the aura) that usually precede the headache phase of an attack.

The pathogenesis and pathophysiology of migraine are poorly understood. A diverse group of variables have been implicated in the pathophysiology of migraine, in particular, the serotoninergic system, with drugs that release serotonin shown to precipitate migraine attacks [3], while drugs that interact with serotonin receptors have beneficial prophylactic and abortive effects [4]. Glutamate, which is a major excitatory neurotransmitter in the central nervous system, has also been broadly involved in migraine pathophysiology. Altered glutamate levels have been measured in migraine patients [5] and glutamate has been implicated in trigeminal activation and cortical spreading depression (CSD), [5]. In fact, stimulation of the trigeminovascular system may be responsible for the pain process during a migraine episode, whereas CSD seems to underlie the aura symptoms [6]. More specifically, CSD activates the afferences from the trigeminal system, which provokes an inflammation of meninges underlying the pain and causing the headache [7]. Another neurotransmitter that plays an important role in migraine pathophysiology, Gamma-aminobutyric acid (GABA) [8]. GABA, which acts mainly via GABA A and B receptors, is the main inhibitory neurotransmitter in the brain. Catecholaminergic, serotoninergic and glutamatergic neurons are all under GABAergic inhibitory control. GABAergic anticonvulsivant medications, are a first line of therapy for prevention for migraine [9]. Baclofen, a GABA B analog, has also been shown to have an antinociceptive effect in the central nervous system, and thus to be efficient in the prophylaxis of migraine [10]. Furthermore, GABA A receptor agonists (Sodium valproate, gabapentin, topiramate) are currently employed in preventing migraine or reducing the frequency and the duration of attacks [11,12].

The GABA A receptor is a member of a superfamily consisting of pentamers of homologous subunits arranged around a central ion conducting channel (Cl- ions) [13]. There are 19 different subunit genes, divided into eight subunit classes: β1–3, θ, η1–2, δ, π, α1–6, γ1–3, ε. GABA A receptors are the targets of sedating drugs, such as benzodiazepines, barbiturates, neurosteroids, and ethanol [14].

**Figure 1:**
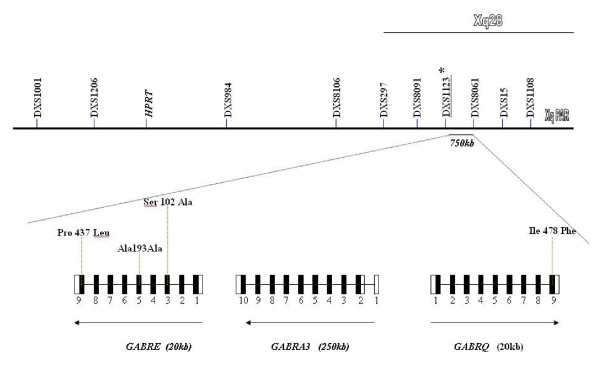
**Schematization of the location of the 4 studied SNPs on the chromosome X (q28)**. Pro 437 Leu is a new variation identified by sequencing of exon 9 of GABRE gene.

While the mode of transmission of migraine is broadly believed to be multifactorial, a role for a major susceptibility gene has been postulated. In fact, migraine shows strong familial aggregation. Approximately 50% of migraine sufferers have an affected first degree relative, with familial incidence figures varying from 61% to 90% [15]. Two Danish population based survey have provided evidence to suggest that MA and MO may be two distinct disorders with an independent genetic identity [16,17]. However, results of Australian [18] and Dutch [19] studies have suggested that migraine with and without aura are not etiologically distinct. It is most likely that there is at least some shared genetic liability between the two subtypes. Genetic characterization of the migrainous disorder is making steady progress with an increasing number of genomic susceptibility loci now identified on chromosomes 1q, 4q, 5q, 6p, 11q, 14q, 15q, 17p, 18q, 19p and Xq and more specifically in the Xq 24–28 region [20,21]. This region contains a cluster of GABA A receptor subunit (epsilon, alpha 3, theta) genes. A cluster of genes, GABRE-GABRA3-GABRQ, spans a genomic region of about 700 Mb (figure 1); GABRE and GABRA3 genes encode for epsilon and alpha3 subunits respectively and are transcribed in centromeric direction, whilst the GABRQ gene encodes for the theta subunit and is transcribed in telomeric direction. Interestingly, studies performed in rat demonstrate that these three subunits are co-expressed in specific brain regions, in particular the locus caeruleus, with studies demonstrating this may be involved in the migraine disorder [22].

In the present study, we investigated three Single Nucleotide Polymorphisms (SNPs), previously described (rs 2050843 and rs 22566882 in GABRE gene and rs 3810651 in GABRQ) and a new mutation in exon 9 of the GABRE gene, which has been recently identified by sequencing. These polymorphism studies were undertaken in a large Australian population of unrelated subjects involving 275 migraineurs and 275 matched controls.

## Methods

### Subjects

A cross-sectional association approach was employed, utilizing genomic DNA samples obtained from 275 migraine affected individuals and 275 controls. The populations consisted of Caucasians from the general Australian Community. Before commencing the study, ethical clearance was sought and approved by Griffith University's Ethics Committee for Experimentation on Humans. Individuals for the study were recruited from the local general population using advertising via notices at Doctors Surgeries and in Pharmacies, as well as through media release on local radio, television and in press articles. Potential participants contacted the Genomics Research Centre and suitability for inclusion in the study was determined using a detailed questionnaire completed by all participants, providing demographic parameters, ancestry information and family medical history. The control group consisted of individuals with no family history of migraine. Volunteers who did not meet these criteria were not included in the study. All recruited individuals for the study gave informed consent and were adult (18 years or older) Caucasians of European descent living in Australia, having emigrating ancestors within the last 160 years from various locations within the British Isles and other parts of Europe. In total ~600 cases and an equivalent number of controls were collected over several years, with a random 275 cases and 275 matched controls used routinely for our genotyping studies, and other samples set aside for future independent studies. Samples used for the genotyping studies were all individuals, not families, with care taken not to include any related individuals in the case-control population. Case and control individuals were recruited from in and around the South Eastern Australia Region, with collections undertaken in the Genomics Research Centre Clinic at the Gold Coast, Queensland, Australia. To minimize potential bias from population stratification, the control group was matched for sex, age (+/- 5 years) and ethnicity. Migraine patients were clinically defined and suitably matched with non-migraine individuals who made up the control population. The subjects were diagnosed for migraine by a clinical neurologist using a detailed questionnaire in accordance with the International Headache Society criteria [2]. Questions used to define migraineurs included length and frequency of attack; pain location, type and intensity; associated symptoms such as nausea, vomiting, phonophobia, photophobia and other visual disturbances, and other neurological symptoms. All individuals were grouped together and phenotyped as being affected with typical migraine (MA+ MO = Migraine), as well as being diagnosed separately as MA or MO subgroups. The blood samples obtained from patients were collected through the Genomics Research Centre patient clinic and purified DNA from these samples was obtained using standard extraction methods. Around 90% of the examined DNA samples gave good genotyping results for the 4 selected genetic markers. We excluded the samples with unclear genotyping results. The study protocol was approved by Griffith University's Ethics Committee for Experimentation on Humans.

### Markers/genotyping

The study investigated three different polymorphisms at the GABRE gene locus. The first marker was located in exon 3 of GABRE and named GABRE 3 for the study. GABRE 3 is a non synonymous SNP (ref database, rs 2050843) at position 102 (G→T). PCR reactions (10μl final volume) containing 2 mmol/L MgCl_2_, 0.8 mol/L of each primer, 200 mol/L dNTPs, 1 unit of *Taq *polymerase and approximately 20 ng of genomic DNA were undertaken for genotyping purposes.

Primers were:

Sense: 5'-TAGATGCTGAAACTGTGTGG-3'

Anti sense: 5'-AAATCCCTTTCTCCCTCCAG-3'

Thermal cycling was performed with an initial denaturation of 60 seconds at 94°C, followed by 35 cycles of 30 sec at 94°C, 30 sec at 58°C, 30 sec at 72°C, and a terminal extension of 10 min at 72°C. PCR products were then digested with *Bgl II *and analyzed by electrophoresis on 4% agarose gels. Ethidium bromide stained gels were digitally imaged and manually scored for genotypes. The PCR products were 327 bp in size. The GABRE 3 G alleles did not digest with *Bgl II*, whereas T alleles digested to give 177 bp and 150 bp fragments.

The second marker was a synonymous SNP (ref SNP database, rs 2256882) at position 193 (C → T) in exon 5, named GABRE 5. PCR reactions and thermal cycling conditions were the same as those cited above.

Primers were:

Sense: 5'-GACTCTGGCCATTCATTGGT-3'

Anti sense: 5'-TGGCAGGAAAGGAAATGAGG-3'

To detect this SNP, PCR products were digested with *Hpa II *and analyzed by electrophoresis on 4% agarose gels. Ethidium bromide stained gels were digitally imaged and manually scored for genotypes. The PCR products were 323 bp in size. The GABRE 5 T alleles did not digest with *Hpa II*, whereas C alleles digested to give 180 bp and 143 bp fragments.

The last marker for GABRE gene was a new variation identified by sequencing exon 9 and identified at position 437 (C → T), as a non synonymous SNP named GABRE 9. PCR reactions were again the same as those cited above.

Primers were:

Sense: 5'-ATGGTGAGAAGTTGGAGGAG-3'

Anti sense: 5'-AGAGGGGCAGCAAAGACAAA-3'

Thermal cycling was performed with an initial denaturation of 60 seconds at 94°C, followed by 35 cycles of 30 sec at 94°C, 30 sec at 60°C, 30 sec at 72°C, and a terminal extension of 10 min at 72°C. PCR products were then digested with *Ava II *and analyzed by electrophoresis on 2.5% agarose gels. Ethidium bromide stained gels were digitally imaged and manually scored for genotypes. The PCR products were 683 bp in size. The GABRE 9 T alleles did not cut with *Ava II *and gave three fragments of 372 bp, 190 bp and 121 bp, whereas C alleles gave 301 bp, 190 bp, 121 bp and 71 bp fragments.

The last marker studied was a synonymous SNP (ref SNP database, rs 3810651) at position 1432 (A → T) in exon 9 of GABRQ gene, named GABRQ. PCR amplicons of 300–500 nt containing the 5' prime region and all 9 exons of the GABRQ gene, including intron/exon boundaries, were designed using the Oligo 4.0 software.

Primers were:

Sense: 5'-TCTCTCCCCATCACCCCAGC-3'

Anti sense: 5'-TTCGACACGGTTGCGGATTT-3'

PCR conditions were as follows; 1.5 mM of MgCl2, 1 × standard PCR buffer, 0.2 mM of dNTPs, 0.5 μM each of forward and reverse primers, 1 unit of Taq polymerase, 40 ng of genomic DNA, mixed to final volume of 25 μl with sterile distilled water. The thermal cycle parameters included: 1 cycle at 95°C for 3 min for an initial denaturation, followed by 35 cycles of denaturation for 30 sec at 94°C, primer annealing for 30 sec at TM, primer extension for 45 sec at 72°C and a final extension for 10 min at 72°C. Samples were then exosap digested (Amersham) and sequenced using the Big Dye Terminator Ready Reaction Kit (Applied Biosystem). Sequencing reactions were performed on a 9700 Thermal Cycler (Applied Biosystems) for 25 cycles of 95°C for 10 sec, TM for 5 sec and 60°C for 2 min. After the sequencing, each reaction was column purified (Amersham) to remove excess dye terminators. Sequencing of the products was performed on the ABI prism 3100 Genetic Analyser (Applied Biosystems). Polymorphisms were detected by multiple alignments of sequences using the program Autoassembler (Applied Biosystems).

### Statistical analysis

To detect association between each marker and migraine, we performed chi-square (χ^2^) analysis to test for significant differences in allele and genotype frequencies in case versus control results [23]. χ^2 ^provides the likelihood of a deviation in the distribution of the same attributes in different classes (e.g. allelic frequencies in controls versus affected subjects). If the probability (*P*-value) of an equal distribution between the two groups is below a determined significance level α (in percent), the statistical output will show enough significance to assume LD and therefore association.

We performed χ^2 ^analysis for MA, MO and combined migraine groups versus control subjects for the GABRE 3, 5, 9 and GABRQ polymorphisms. We also tested for linkage disequilibrium between tested markers using Pearson's test to analyze dense genetic maps [24]. The R2 value 0.0 suggests independent assortment, whereas 1.0 means that all copies of the rarer allele occur exclusively with one of the possible alleles at the other marker [25].

In total, we compared each of the three markers in controls with three different case groups of the population (MA, MO, Migraine (MO+MA)). Results were also tested for Hardy-Weinberg Equilibrium (HWE) investigating genotype frequencies of the studied markers to detect a deviation from the normal genotype distribution in the population and odds ratios were calculated to characterize the distribution of distinct genotypes in different phenotypic subgroups of the population. A priori power analysis suggested that if any of the GABRE polymorphisms were to confer an 2-fold or greater difference in odds for migraine the total case and control groups used in this study were of sufficient size to have >90% power to detect an allelic association and >80% power to detect a genotypic association at the 0.05 level.

### Ethical approval

This research was reviewed and approved by the Griffith University Human Research Ethics Committee (ethics protocol number MSC/05/05/HREC) and all subjects participating in the study gave informed consent.

## Results

Three markers located within 15 kb of the coding region of the GABRE gene and a marker in the GABRQ gene were analyzed for association with migraine in a large population (275 migraineurs versus 275 healthy individuals) of Australia Caucasians. The distribution of GABRE 3, 5, 9 and GABRQ genotypes in the studied population did not deviate significantly from Hardy-Weinberg Equilibrium (P > 0.05).

Table 1 represents the results of the allelic and genotypic frequency distribution of GABRE 3. There was no significant association between both allelic and genotype frequencies of GABRE 3 and migraine (χ^2 ^= 0.33, P = 0.56 and χ^2 ^= 0.73, P = 0.695 respectively). Also, allelic frequencies in control groups (pG = 0.762 and pT = 0.23) are comparable to the frequencies reported previously in a Caucasian population (pG = 0.692 and pT = 0.308) [26].

**Table 1:** Distribution of the GABRE 3 mutation (Ser102Ala), GABRE 5 mutation (Ala 193Ala), in migraineurs and controls of original sample (MO migraine without aura, MA migraine with aura).

	***N***	***Alleles***			***Genotypes***	
***Group***	***(alleles)***	***G***	***T***	***GG***	***GT***	***TT***
***GABRE3 SNP***	***rs 2050843***					
Migraine	384	282 (73.4%)	102 (26.6%)	114 (59.4%)	54 (28.1%)	24(12.5%)
Male	112	84 (75%)	28 (25%)			
Female	272	198 (73%)	74 (27%)	72 (53%)	54 (39.7%)	10 (7.3%)
MA	254	194 (76.4%)	60 (23.6%)	77 (60.6%)	40 (31.5%)	10 (7.9%)
MO	130	88 (67.7%)	42 (32.3%)	37 (57%)	14 (21.5%)	14 (21.5%)
Control	374	285 (76.2%)	89 (23.8%)	119 (63.6%)	47 (25.2%)	21 (11.2%)
Male	90	60 (67%)	30 (33%)			
Female	284	225 (79.2%)	59 (20.8%)	89 (62.6%)	47 (33%)	6 (4.4%)
Total case vs control		X^2 ^= 0.33	P = 0.56		X^2 ^= 0.73	P = 0.695
Subtype MA vs cont:		X^2 ^= 0.00	P = 0.99		X^2 ^= 2.08	P = 0.35
Subtype MO vs cont		X^2 ^= 3.63	P = 0.06		X^2 ^= 4.3	P = 0.11
	***N***	***Alleles***			***Genotypes***	
***Group***	***(alleles)***	***T***	***C***	***TT***	***TC***	***CC***
***GABRE5 SNP***	***rs 2256882***					
Migraine	332	34 (10.3%)	298 (89.7%)	6 (3.4%)	22 (12.4%)	149 (84.2%)
Male	92	10 (10.9%)	82 (89.1%)			
Female	262	24 (9.2%)	238 (90.8%)	1 (0.8%)	22 (16.8%)	108 (82.4%)
MA	204	19 (9.3%)	185 (90.7%)	3 (2.9%)	13 (12.8%)	86 (84.3%)
MO	152	16 (10.5%)	136 (89.5%)	3 (3.9%)	10 (13.2%)	63 (82.9%)
Control	374	45 (12%)	329 (88%)	10 (5.3%)	25 (13.4%)	152 (81.3%)
Male	104	12 (11.5%)	92 (88.5%)			
Female	270	33 (12.2%)	237 (87.8%)	4 (3%)	25 (18.5%)	106 (78.5%)
Total case vs control		X^2 ^= 0.57	P = 0.45		X^2 ^= 0.95	P = 0.62
Subtype MA vs cont:		X^2 ^= 0.99	P = 0.32		X^2 ^= 0.94	P = 0.62
Subtype MO vs cont		X^2 ^= 0.24	P = 0.62		X^2 ^= 0.23	P = 0.89
	***N***	***Alleles***			***Genotypes***	
***Group***	***(alleles)***	***T***	***C***	***TT***	***TC***	***CC***
***GABRE9 SNP ***						
Migraine	316	20 (6.3%)	296 (93.7%)	7 (4.4%)	6 (3.8%)	145 (91.8%)
Male	88	6 (6.8%)	82 (93.2%)			
Female	228	14 (6.1%)	214 (93.9%)	4 (3.5%)	6 (5.3%)	104 (91.2%)
MA	198	9 (4.5%)	189 (95.5%)	3 (3%)	3 (3%)	93 (94%)
MO	118	11 (9.3%)	107 (90.7%)	4 (6.8%)	3 (5.1%)	52 (88.1%)
Control	280	23 (8.2%)	257 (91.8%)	7 (5%)	9 (6.4%)	124 (88.6%)
Male	80	4 (5%)	76 (95%)			
Female	200	19 (9.5%)	181 (90.5%)	5 (5%)	9 (9%)	86 (86%)
Total case vs control		X^2 ^= 0.79	P = 0.37		X^2 ^= 1.16	P = 0.56
Subtype MA vs cont:		X^2 ^= 2.50	P = 0.11		X^2 ^= 2.06	P = 0.36
Subtype MO vs cont		X^2 ^= 0.13	P = 0.72		X^2 ^= 0.36	P = 0.83

As shown in Table 1, the synonymous GABRE 5 marker distribution for allelic and genotype frequencies did not present significant association for migraine (χ^2 ^= 0.57, P = 0.45 and χ^2 ^= 0.95, P = 0.62 respectively for allelic and genotype frequencies). Similarly to the previous marker, the allelic frequencies for GABRE 5 found in our population (pC = 0.88 and pT = 0.12), are similar to prior reports (pC = 0.91 and pT = 0.08) in a European population [27].

The analysis of the non synonymous GABRE 9 marker also did not show any significant association with migraine (χ^2 ^= 0.76, P = 0.37 and χ^2 ^= 1.16, P = 0.56 respectively for allelic and genotype frequencies).

Stratified analyses of migraine subtypes was also undertaken but did not indicate any association specifically attributed to the MA or MO subtype group for either allelic or genotypic frequencies for all of the three studied SNPs (P > 0.05). Similarly, when we analyzed by gender, no significant association was observed for the three GABRE genotype and allelic distributions (P > 0.05) (cf. Table 1).

Statistical analysis of the GABRQ variant revealed no significant difference between genotyped migraineurs and the matched control group in relation to genotype frequencies (χ^2 ^= 0.57, P = 0.753) and allele frequencies (χ^2 ^= 0.19, P = 0.664) (cf. Table 2). Furthermore, no significant difference was seen when the migraine population was subdivided into MA and MO compared to control group for both allelic and genotypic frequencies (P > 0.05), although the increased frequency of the TT genotype in MO (27%) compared to MA (20%) may warrant follow-up in a larger study group. The results were also not statistically different between the migraine and control groups regarding the gender (P > 0.05). With regard to male allele frequencies, it was however interesting to note a higher frequency of the T allele in male MO migraineurs (60%) compared to the male MA migraineurs (41.2%) and the male control group (44.1%).

**Table 2:** Distribution of the GABRQ gene exon 9 variation (P437I) in migraineurs and controls of original sample (MO migraine without aura, MA migraine with aura).

	***N***	***Alleles***			***Genotypes***	
	***(alleles)***	***A***	***T***	***AA***	***AT***	***TT***
***GABRQ SNP***	***rs 3810651***					
Migraine	350	210 (60%)	140 (40%)	82 (40%)	74 (37%)	46 (23%)
Male	54	28 (51.8%)	26 (48.2%)			
Female	296	182 (61.5%)	114 (38.5%)	54 (36%)	74 (50%)	20 (14%)
MA	208	131 (63%)	77 (37%)	54 (45%)	43 (35%)	24 (20%)
MO	140	77 (55%)	63 (55%)	27 (34%)	31 (39%)	22 (27%)
Control	367	226 (61%)	141 (39%)	94 (44%)	72 (34%)	47 (22%)
Male	59	33 (55.9%)	26 (44.1%)			
Female	309	194 (62.8%)	115 (37.2%)	61 (40%)	73 (47%)	21 (13%)
Total case vs control		X^2 ^= 0.19	P = 0.664		X^2 ^= 0.57	P = 0.753
Subtype MA vs cont:		X^2 ^= 0.11	P = 0.739		X^2 ^= 0.25	P = 0.881
Subtype MO vs cont		X^2 ^= 1.82	P = 0.176		X^2 ^= 2.65	P = 0.265

Linkage disequilibrium analysis was also undertaken between the 3 GABRE and GABRQ markers. The analysis of LD between the GABRE markers revealed a moderate but significant linkage disequilibrium between GABRE 5 and GABRE 9 (R^2 ^= 0.38, P = 0.00001). However, this LD value decreased by more than 30% and become non significant (P ≥ 0.05) when measured between GABRE 3 and GABRE 5 on one hand, and GABRE 3 and GABRE 9 on the other hand (cf. Table 3). The analysis of LD between the 3 GABRE markers and GABRQ did not show any significant linkage disequilibrium (P > 0.05) (cf. Table 3).

**Table 3:** Pearson's correlation between the 3 GABRE and GABRQ genetic markers.

	**GABRE 3**	**GABRE 5**	**GABRE 9**	**GABRQ**
*Pearson correlation (%)*	1	0.133	0.074	0.002
*P value*		0.019*	0.233	0.571
*Pearson correlation (%)*	0.133	1	0.376	0.004
*P value*	0.019*		0.00001**	0.539
*Pearson correlation (%)*	0.074	0.376	1	0.008
*P value*	0.233	0.0001		0.496
*Pearson correlation (%)*	0.002	0.004	0.008	1
*P value*	0.571	0.539	0.496	

* Correlation is significant at the 0.05 level.

** Correlation is significant at the 0.001 level

## Discussion

A substantial body of literature suggests that GABA may be involved in the neuropathophysiology of migraine yet there have been few studies investigating GABA receptor genes as potential candidate genes for migraine. *γ*-Amino butyric acid (GABA) is the major inhibitory neurotransmitter of the brain, occurring in 30–40% of all synapses in regions such as the cerebral cortex, hippocampus, thalamus, basal ganglia, cerebellum, hypothalamus and brainstem [28]. *γ*-Amino butyric acid type A (GABA A) receptors are the major sites of fast synaptic inhibition in the brain and are also the sites of action for many psychoactive drugs including the benzodiazepines and barbiturates [29]. In mammals, they are constructed as pentameric structures from multiple subunits selected predominantly from the following distinct classes: *α *(1–6), *β *(1–3), *γ *(1–3), *δ*, *ε*, *θ *and *π*, creating an incredible (165) potential for structural diversity. Studies have demonstrated that the subunit combination determined the affinity for GABA and the specific effects of allosteric modulators [14].

Recently, new members (epsilon and theta) of the GABA A receptor gene family have been discovered [30-32]. It is worth noting that alpha3-, theta- and epsilon-subunits are clustered on the X-chromosome [33] and that functional expression of recombinant receptors, as well as amino acid sequence identity analysis, have suggested that theta- and epsilon-subunits may substitute for beta- and gamma-subunits, respectively [33,34].

Functional expression of recombinant subunits has indicated that, in some brain areas (i.e. locus caeruleus, dorsal raphe, and hypothalamus) already demonstrated to be potentially involved in the migraine disorder, the theta subunit is co-expressed with epsilon and alpha3 subunits, suggesting that these subunits are associated in some native GABA A receptors [35].

In this study, we analyzed the distribution of genotype and allele frequencies of three SNPs located in the GABRE gene and one variant in the GABRQ gene in a large population of migraineurs and matched controls. Three of these markers are non synonymous SNPs (GABRE 3, GABRE 9 and a new variation in GABRQ) and induce changes of amino acids in the protein receptor (Ala102Ser and Pro437Leu, Ile478Phe respectively). The last SNP studied was synonymous (Ala193Ala), located in exon 5 of the GABRE gene (GABRE 5).

The study did not find any association between the three analysed SNPs in the GABRE gene and migraine. The GABRE gene contains 9 exons, which code for 4 different variants of GABRE [36]. The role of this splicing remains unclear at present. The first variant is a the full-length functional transcript and is highly expressed in heart and lung and also present in brain [37,38]. In variant 2, exons 1–3 are spliced out, and in variant 3 the same splicing occurs in combination with a deletion of residues 127–158 from the centre of exon 4 [36]. The mRNAs of variants 2 and 3 have been found in several peripheral tissues [37]. In variant 4 only exon 1 is deleted. Variants 2, 3 and 4 lack the signal peptide and also (for forms 2 and 3) lack a significant region of the extracellular N-terminal domain, but the roles for these variants (form 2, 3 and 4) in combination with the full-length variant 1 remain unclear.

Our results also did not show any association between the tested GABRQ marker (rs 3810651) and migraine. No significant association was reported for both MA and MO populations for the studied single missense variation, A1432T. Of interest is the frequency distribution of the T allele in male MO (60%) patients versus male MA patients (41.2%) and versus male controls (44%). While this analysis did not reach statistical significance, due to small numbers in the male subgroup, it may warrant further investigation in a larger study group. The A1432T variation altered a conserved amino acid; the isoleucine amino acid is conserved in primate and mouse. The functional significance of the isoleucine residue in codon 478 is unknown but the high degree of conservation across several species argues strongly in favor of an important role of this amino acid in the function of GABRQ protein. The hypothesis that the isoleucine residue in codon 478 may constitute an important point for GABRQ function, which would be perturbed (pathogenic) by the phenylalanine substitution, should be considered.

Previous linkage studies in our research group have reported mapping of a migraine gene to the X chromosome in three large Australian pedigrees [20,39]. Although results from haplotype and linkage analyses (employing 28 markers spanning the entire X chromosome) localized the disease locus in the Xq 24–28 region, these findings may be specific to the studied population, which may not be representative of the general population [20].

The GABA neurotransmitter has been previously implicated in the pathophysiology of migraine, but there have been only recent studies in migraine genetics. Russo *et al*. reported significant linkage, in five Italian families suffering from a migraine subtype with the 15q11–q13 chromosomal region, a region containing clusters of GABA A receptor subunit (beta 3, alpha 5 and gamma 3) genes [40]. More studies need to be undertaken and in particular, on other genetic markers in the other GABA receptors genes present in the Xq28 gene cluster. The GABA A receptor subunit alpha 3 (GABRA3) gene is also located in this region. A dinucleotide cytosine-adenosine repeat polymorphism with 6 alleles representing 11 to 16 repeats has been described by Hicks (2000) in the GABRA3 gene [41]. This polymorphism has been associated with Multiple Sclerosis [42], but also with Bipolar Disorder [43,44]. Both of these neurological disorders shared a comorbidity with migraine [45]. A GABRA3 microsatellite has been also studied in another psychiatric disorder population (suicide attempts) but no significant association has been reported for this genetic marker [46].

GABA levels in the cerebro-spinal fluid of patients during a migraine attack have been reported to be higher compared to the levels measured during a headache free period in the same individuals [47]. Kowa *et al*. (1992), have also observed higher GABA levels in blood platelets of patients suffering from tension headache [48]. Furthermore, the GABAergic migraine prophylactic drugs may restore a normal cortical inhibitory potential by elevating cortical threshold for spreading depression propagation in patients with migraines [49]. GABA neurotransmitter plays a key part in cerebral physiology, and is able to inhibit a number of neurohormones including serotonin, catecholamines and glutamate. Like other ligand gated ion channels, such as the GABA A receptor, the ionotropic glutamate receptor subunits possess four subunits. The ionotropic glutamate receptor, AMPA-selective glutamate receptor 3, has been located to the Xq 24–28 region, mapped migraine susceptibility [50].

## Conclusion

Previous studies in our laboratory have reported significant evidence for the location of a migraine susceptibility locus on chromosome Xq [39] and more specifically to the Xq 24–28 region [20]. Previous candidate genes 5-HT_2C _receptor gene, residing in this region have not revealed genetic differences between migraineurs compared to controls [51-53]. Also, lack of association of Monoamine Oxidase genes located on chromosome X, with migraine susceptibility has been reported in several studies [54-56]. Chromosome X studies and migraine susceptibility have been investigated for the last decade. The prevalence of migraine in children before puberty has been reported to be quite similar in boys and girls (4%) [57] but migraine occurs more frequently in adult women (18%) than in men (6%) [58]. GABAergic neurons are strongly modulated by ovarian hormones with studies showing an effect of estrogen and progesterone and its metabolites on GABA receptors [59-61], as well as cortical GABA levels [62]. Although this study has not implicated tested markers in the GABRE gene, further investigation of other GABA related genes, particularly in the cluster of GABA A receptor subunit genes residing at Xq 24–28 region, is required to define their potential role in migraine.

## Competing interests

The authors declare that they have no competing interests.

## Authors' contributions

FF and TE were responsible for undertaking all the experiments and the analysis of data was undertaken collaboratively by all authors. LG coordinated the study and revised the manuscript. All authors read and approved the final manuscript.

## Pre-publication history

The pre-publication history for this paper can be accessed here:


